# Intelligent Color Vision System for Ripeness Classification of Oil Palm Fresh Fruit Bunch

**DOI:** 10.3390/s121014179

**Published:** 2012-10-22

**Authors:** Norasyikin Fadilah, Junita Mohamad-Saleh, Zaini Abdul Halim, Haidi Ibrahim, Syed Salim Syed Ali

**Affiliations:** 1 School of Electrical & Electronic Engineering, Universiti Sains Malaysia, Engineering Campus, 14300 Nibong Tebal, Pulau Pinang, Malaysia; E-Mails: norasyikin@ump.edu.my (N.F.); haidi@eng.usm.my (H.I.); 2 Collaborative Microelectronic Design Excellent Centre, Universiti Sains Malaysia, Engineering Campus, 14300 Nibong Tebal, Pulau Pinang, Malaysia; E-Mail: zaini@cedec.usm.my; 3 Felda Agricultural Services Sdn Bhd, Pusat Perkhidmatan Pertanian Tun Razak, Beg Berkunci No. 3, 26400 Bandar Jengka, Pahang Darul Makmur, Malaysia; E-Mail: syedsalim.sa@felda.net.my

**Keywords:** artificial neural network, principal component analysis, digital image processing, oil palm fresh fruit bunch

## Abstract

Ripeness classification of oil palm fresh fruit bunches (FFBs) during harvesting is important to ensure that they are harvested during optimum stage for maximum oil production. This paper presents the application of color vision for automated ripeness classification of oil palm FFB. Images of oil palm FFBs of type DxP Yangambi were collected and analyzed using digital image processing techniques. Then the color features were extracted from those images and used as the inputs for Artificial Neural Network (ANN) learning. The performance of the ANN for ripeness classification of oil palm FFB was investigated using two methods: training ANN with full features and training ANN with reduced features based on the Principal Component Analysis (PCA) data reduction technique. Results showed that compared with using full features in ANN, using the ANN trained with reduced features can improve the classification accuracy by 1.66% and is more effective in developing an automated ripeness classifier for oil palm FFB. The developed ripeness classifier can act as a sensor in determining the correct oil palm FFB ripeness category.

## 1. Introduction

Quality is the most important factor for agricultural and food products because high quality products are significant for success in today's highly competitive market. In agricultural applications, the quality of a product—especially fruits—is often classified by their texture, shape and color. These features are usually observed using human's vision particularly in determining the ripeness of fruits. However, the method of human grading is tedious and may be erroneous. This leads to extensive researches on automated fruit grading using sensor-based technologies such as image sensors. It is believed that the use of non-contact image sensing technology combined with robust computing and decision processes provides automated, non-destructive and cost-effective method to determine the quality of agricultural and food products [1].

Oil palm fruit is one of the major agricultural products particularly in Malaysia. It produces palm oil, which is the basic ingredient in manufacturing of soaps, candles, margarine, shortenings, domestic frying oil and snack food. An oil palm fresh fruit bunch (FFB) may contain up to 2000 fruits with an individual weight of 3-30 g and 2-5 cm in size [2,3]. Oil palm FFB of type *Elaeis guineensis* is common in Malaysia. The fruit color varies from very dark purple to orange depending on its gene and ripeness. The oil content for different stages of FFB ripeness also varies, and it is generally stated as oil-to-bunch ratio [2]. As the oil content of FFB is a function of its degree of ripeness [4], it is crucial that the FFBs are harvested at the optimum ripeness.

According to [2], it is practical to observe the number of loose fruits on the ground to determine whether the FFB on the tree is ripe. Hitam and Yusof [4] discussed two methods of expressing the number of loose fruits. One of them is the number of loose fruits on the ground before the FFB is cut, and the other is the number of loose fruit sockets on the bunch. The latter is feasible for short trees since the harvester could clearly see the FFB, while the former method is often used for tall trees. The observation of loose fruits for ripeness prediction of oil palm FFB on tall trees has been practiced until today. However, this method may be inaccurate because loose fruits might fall under a different tree and can be stuck in the fronds, washed away by heavy rain, or taken by animals in the estate. The probability of harvesting FFBs that are not ripe will increase. Moreover, this method is time consuming and laborious, which could lead to higher harvesting and production costs.

Malaysian Palm Oil Board (MPOB) has established fifteen classes of FFB in the grading of oil palm in palm oil mills: ripe, underripe, unripe, overripe, empty, rotten, long stalk, unfresh/old, dirty, small, pest damaged, diseased, dura, loose fruit, and wet [5]. Most studies focused on the grading of two, three or four ripeness stages: unripe, underripe, ripe and overripe. These studies employed color vision inspection in assessing fruit ripeness. For example, MPOB identified purplish black fruits as unripe, reddish black as underripe, red as ripe, and reddish orange as overripe [6].

Images in red, green and blue (RGB) color space were used to analyze the color of oil palm fruits. Ismail *et al.* [7] found that the ranges of color intensity for all ripeness categories were almost the same. The only attribute that could differentiate between ripe category and others was the average of red value. However, a later study by Ghazali *et al.* [8] discovered that the red components for unripe and underripe categories were almost the same. Thus, red component was not able to distinguish between unripe and underripe categories, and could not be an attribute for ripeness classification.

The optimum ripeness of oil palm FFB is indicated by the amount of oil extracted from the fruit bunch. Therefore, Choong *et al.* [9] investigated the correlation between the color of oil palm fruits and their oil content. It was reported that there was a positive correlation between both attributes. Underripe fruit has the lowest oil content, ripe fruit has the highest oil content and the oil content deteriorates when the fruit reached overripe stage. Similar results were also reported in other studies of correlation between the color of oil palm FFBs and their oil contents [10–12]. Hudzari *et al.* [11] studied the relationship between FFB color, light intensity, and oil content for three ripeness stages; unripe, ripe and overripe. They found that as light intensity becomes higher, the RGB pixel values increase. Thus, they converted RGB image to Hue, Saturation, and Intensity (HSI), and constructed a hue histogram [13–15]. Tan *et al.* discovered that in four different sides of an FFB, there were differences in dominant hue peaks [14]. This could be due to the uneven color of the FFB that resulted from different amount of exposure to sunlight. However, statistical evaluation showed that there was a good correlation coefficient between the dominant hue peak and the FFB oil content. Ismail *et al.* reported a high correlation between hue value and oil content [15]. They conducted an experiment with FFB that were on trees and concluded that unlike RGB intensities, hue value was not affected by the variances of lighting intensity. Therefore, hue value is an important attribute for detecting FFB color in any light intensity.

Principal component analysis (PCA) has been widely used to accomplish the task of pattern recognition or data reduction for multivariate data [16,17]. For instance, in a work to develop a classifier for polarimetric synthetic aperture radar images, Zhang and Wu implemented PCA to reduce 19 features from an image to 13 features. These features were then used for a two-hidden-layer back-propagation neural network for classification [17]. In oil palm study, Junkwon *et al.* [18] used PCA and Euclidean distance to identify four ripeness classes of oil palm FFB. Three features represented by three RGB values were analyzed using PCA to obtain a plot of two principal components. From the plot, four centroid values that indicated four ripeness classes were identified, and the Euclidean distances between the centroid values and the plot of other samples were used to classify the oil palm FFB. This method yielded 75% correct classification for RGB images.

Jamil *et al.* and May and Amaran developed intelligent oil palm FFB grading by using neuro-fuzzy and fuzzy logic, respectively. Jamil *et al.* [19] trained RGB values for 45 FFB images by using Hebb algorithm to identify the best-fit value to represent RGB color of FFB images. Then the color classification was conducted in four steps: fuzzification, rule evaluation, aggregation of the rule outputs and defuzzification. The neuro-fuzzy techniques yielded 73.3% correct classification. Meanwhile, May and Amaran [20] developed an automated oil palm fruit grading system using fuzzy logic algorithm, which gave 86.67% correct classification. More studies of different classification techniques can be done to enhance the classification accuracy of automated oil palm FFB system. For instance, artificial neural network (ANN) classifiers have been successfully implemented for various classification tasks of other different agricultural products. These include classification of the quality for San-Fuji apples, cherries, Iyokan oranges, and beans [21].

The objective of this work is to develop an algorithm for automatic intelligent grading of oil palm FFB based on color vision in a natural light environment. The color measurement was based on hue distribution of oil palm FFB image. The feasibility of applying PCA for data reduction and ANN intelligent system for oil palm FFB ripeness classification were investigated. The results were presented and discussed.

This paper consists of four sections. Current oil palm harvesting methods and previous studies involving color vision for ripeness classification of oil palm FFB are discussed in this section. Section 2 presents the setup of the proposed classification system and explains the steps involved in developing an oil palm FFB ripeness classifier. Then the ripeness classification results obtained are presented and discussed in Section 3. Lastly, Section 4 concludes the findings of the research work.

## 2. Materials and Methods

**Figure 1.**
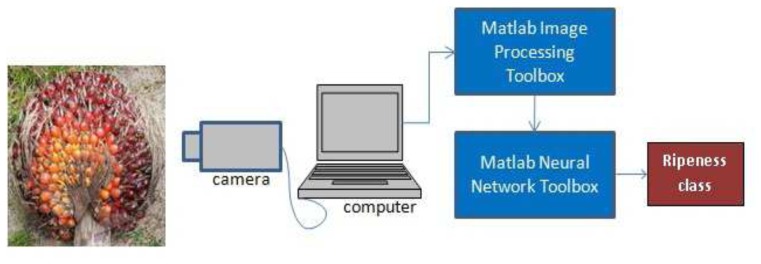
Oil palm FFB grading system.

The intelligent grading system consisted of a camera for image acquisition and a computer for data storage, image pre-processing and ANN classification. The system is illustrated in Figure 1. A Vivotek IP8332 Network Bullet Camera (0 Lux, 1.0 M pixels, F1.8) was used to acquire the image of oil palm FFB. This camera was chosen due to its ability to adapt to constantly changing outdoor lighting conditions. Matlab Image Processing Toolbox was used to process each captured image. An ANN system was trained and tested using the Matlab Neural Network Toolbox.

Oil palm FFBs of type DxP Yangambi were sourced from Felda Agricultural Services Sdn. Bhd. (FASSB). For this work, researchers managed to get 80 FFBs cut-off, with equal numbers for each ripeness class. A FASSB's trained grade inspector segregated the fruits into four ripeness categories: unripe, underripe, ripe and overripe. Rotten, empty and infected FFBs were discarded. At most, four color images were captured for each FFB at different areas of the bunch. The total images taken from 80 FFBs were 208 images. Each image was at the size of 480 × 640 in a 24-bit RGB format. All the captured images were stored in a computer for further processing. Then the images were randomly divided into 3 sets; 120 images were categorized as the training set, 28 images were grouped as the validation set, and 60 images formed the independent test set. No more than 60 images could be used as test set due to the limited number of FFBs that could be obtained.

**Figure 2.**
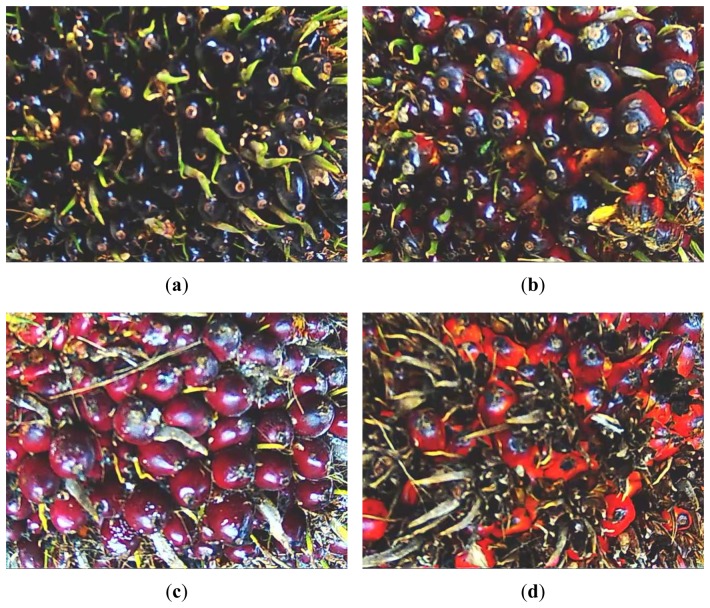
Oil palm FFB images for four ripeness categories: (**a**) Unripe; (**b**) Underripe; (**c**) Ripe; (**d**) Overripe.

Four sample images from different ripeness categories are shown in Figure 2. It can be seen that the unripe fruits are in deep violet to black. As the fruits ripen, they turn red. The overripe oil palm FFB shows that most of the outer fruits are gone, and the inner fruits are orange in color.

Secondly, the images were processed using digital image processing technique to obtain color features of the fruits. In this method, the images were segmented into two parts, which were fruits area and spikes area. This segmentation process is further clarified in Section 2.1.

Thirdly, after the fruits area was obtained for each image, color features were extracted. In this work, hue for each fruit pixel was calculated and a hue histogram representing the feature vector for each image was obtained. This feature vector represented the parameter for ripeness classifier. A detailed explanation of color feature extraction is explained in Section 2.2.

Lastly, an ANN classifier was developed to classify the ripeness of oil palm FFB. Two methods were investigated; one of them used all features as the input parameters, whereas the other used reduced PCA features as the input parameters. These methods are clarified in Section 2.3.

### 2.1. Image Segmentation

Image segmentation is a process that divides the image into regions [22]. As seen earlier in Figure 2, there are two distinct regions in each of the oil palm FFB images, which are spikes and fruits. The fruits region was of interest in this research. Therefore, the image was partitioned into two regions to obtain the fruits region.

**Figure 3.**
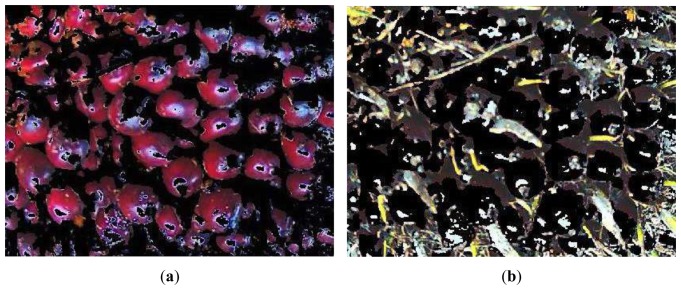
Segmented images of oil palm FFB's (**a**) fruits and (**b**) spikes.

In this work, oil palm FFB images were segmented based on clustering method used by Jaffar [12]. K-means clustering was implemented for image segmentation in L*a*b* color space. At first the RGB image was converted into two-dimensional image with a* and b* color planes. Then the most representative number of clusters was determined for each oil palm FFB category, so that each cluster represented either the fruits or the spikes by trying with 2 to 5 clusters. Three clusters were found to be sufficient to distinguish between the spikes and the fruits. Hence, the mean values of three colors were obtained from each image based on different ripeness category of oil palm FFB. From all the images, five discriminating color mean values were identified. These values were used as color markers to classify every pixel in an image by calculating the Euclidean distance between each pixel and each color marker. The smallest distance indicated that it closely matched the color marker. In this process, a binary mask image was formed to obtain the fruits' segmented image; the fruits' pixels were labeled as “1” and the spikes' pixels were labeled as “0”. After that, the binary mask was conceptually placed on top of the original RGB image by multiplication to produce a segmented image. Examples of segmented images are shown in Figure 3.

### 2.2. Color Features Extraction

Color is an important feature in determining the ripeness of an oil palm FFB. Compared with RGB or CIExy values, hue measurement has shown to be a good discriminator for oil palm colors [23]. Thus, in this work, the RGB image was converted to HSI color model to extract the hue values. This color model has been an ideal tool in depicting humans color interpretation. Mathematically, the hue value *H* is given by [22],

(1) $$H=\{\begin{array}{rrr} \cos^{-1}\frac{\frac{1}{2}[(R-G)+(R-B)]}{{\left[{\left(R-G\right)}^{2}+(R-B)(G-B)\right]}^{1/2}} & \text{if} & B\leq G\\ 360-\cos^{-1}\frac{\frac{1}{2}[(R-G)+(R-B)]}{{\left[{\left(R-G\right)}^{2}+(R-B)(G-B)\right]}^{1/2}} & \text{if} & B>G \end{array}$$

where *R*, *G* and *B* are the red, green and blue components of the RGB image for fruits region, respectively. After obtaining the hue values, a hue histogram of 100 bins was obtained. The histogram distribution was indicated as a feature vector for each image. Therefore, for the whole dataset with *N* samples and *q* hue values, a matrix ***X*** of *N*×*q* was obtained as,

(2) $$X=\left(\begin{array}{cccc} x_{11} & x_{12} & \cdots & x_{1q}\\ x_{21} & x_{22} & \cdots & x_{2q}\\ \vdots & \vdots & \ddots & \vdots \\ x_{N1} & x_{N2} & \cdots & x_{Nq} \end{array}\right)$$

In this work, out of 100 bins that represented the hue values, only 59 values represented the color of fruits. These values were used as the features for ANN inputs.

### 2.3. Development of Oil Palm FFB Intelligent Ripeness Classifier

ANN has been widely used to map input patterns with their desired outputs. Its application is wide, ranging from data classification to data prediction and data visualization [24–26]. No pre-defined rules needed to be set for an ANN, as it is able to learn and generalize from “experience” or a set of presented examples [27]. The set of examples is called a training set.

**Figure 4.**
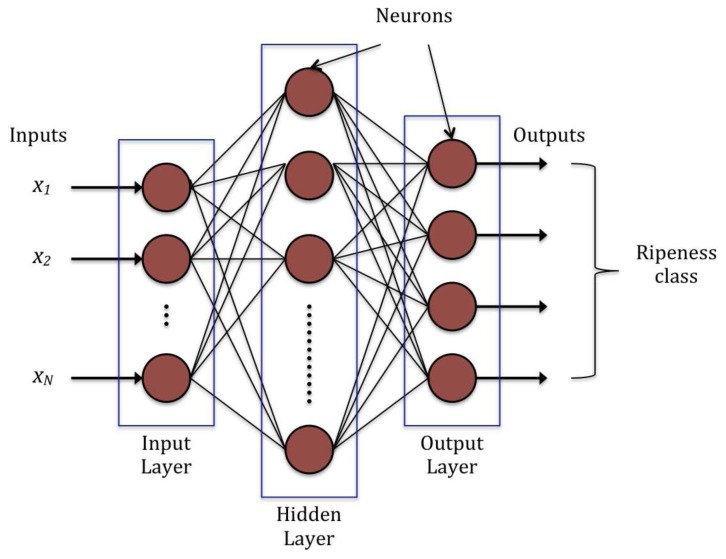
Structure of MLP neural network.

In this work, the employment of multilayer perceptron (MLP) neural network—a commonly used ANN architecture—as the ripeness classifier was investigated. A MLP neural network usually consists of three different layers: input layer, hidden layer and output layer. Each layer comprises a number of neurons, which are also known as processing elements (PE). Detailed descriptions of MLP were documented elsewhere [27–29]. Figure 4 shows the structure of a three-layer MLP architecture. The PEs in the input layer of an MLP does not compute any process. They only buffer the input signals *x*_i_ to the PEs in hidden layer. In the hidden layer, each PE sums up the products of input signals *x*_i_ with their weighted connections *W*_ji_. Mathematically,

(3) $$net_{j}=\sum_{i=1}^{n}W_{ji}x_{i}$$

where *net_j_* is the output for *j*th PE. *net_j_* is then further processed to produce a new output *y_j_* by the following equation;

(4) $$y_{j}=f(net_{j})$$

where *f* is the activation function that determines the processing inside each PE. The output of PEs in the output layer is computed similarly as Equation (4). In this work, the logistic sigmoid (logsig), hyperbolic tangent sigmoid (tansig) and linear (purelin) functions were selected. Respectively, these functions are as given below,

(5) $$f(s)=\frac{1}{1+e^{-s}}$$

(6) $$f(s)=\frac{e^{s}-e^{-s}}{e^{s}+e^{-s}}$$

(7) f(s)=s

To determine the most optimum MLP model, various combinations of transfer functions for hidden and output neurons as listed in Table 1 were applied. The number of output neurons represented the output coding for the ripeness class of oil palm FFB. For CA to CD combinations, each output neuron value was represented in binary, “1” or “0”, whereas for CE and CF, each neuron was represented either as “1”, “2”, “3” or “4”. The representations of the output codings for all ripeness classes are given in Tables 2–4.

The number of optimum hidden neurons was determined experimentally from training processes of the MLP classifiers. The MLP neural network training started with having only one hidden neuron and its performance was recorded. Then, the number of hidden neurons in the MLP was incrementally added, one at a time until there was no longer improvement in the MLP performance. This is known as the network growing approach. In this work, 15 hidden neurons were found to give optimum performance.

All of the MLP networks based on the combinations of transfer functions shown in Table 1 were trained, validated and tested. In a training process, MLP network kept updating the trained-weights in the input and hidden layers after every training cycle to improve its performance. The validation set was used to validate MLP performance by terminating the training process when there was no improvement in the validation performance based on the validation set. The best-performed MLP model was selected based on the highest classification accuracy (*h.c.a.*) of the test set obtained from the percentage of *n* correct classification in the set of *N* test data, using,

(8) $$h.c.a.=\text{max}[(\frac{n}{N_{\text{test}}})\times 100\%]$$

**Figure 5.**
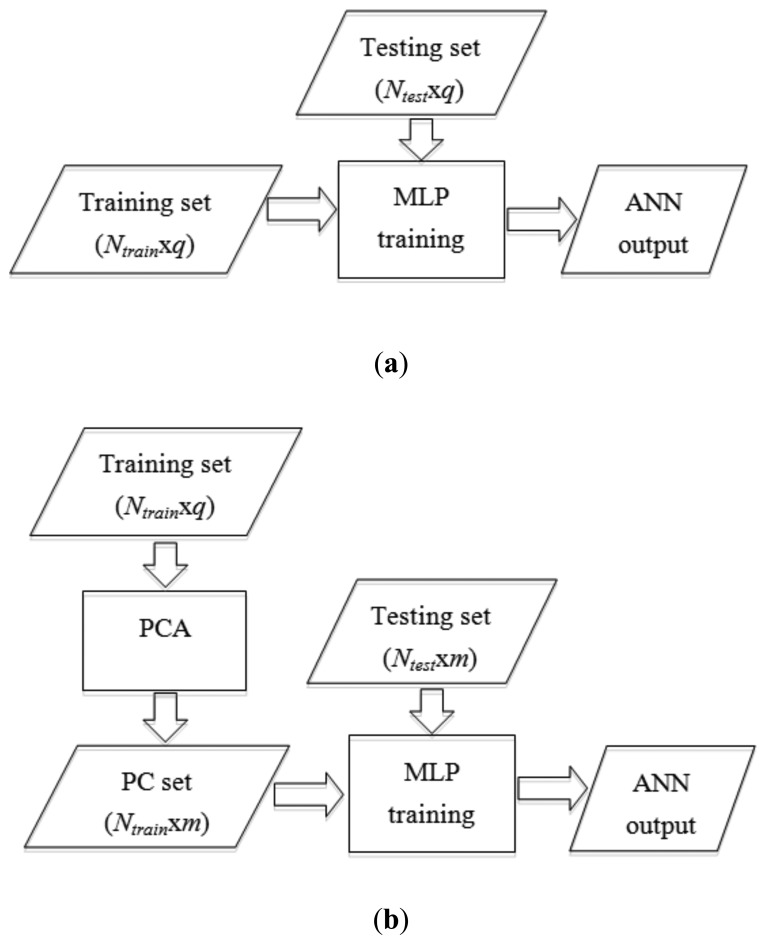
Two experimented methods. (**a**) Method MA; (**b**) Method MB.

To develop an optimum intelligent ripeness classifier, two methods were experimented for each MLP combination and were then compared. The first method, MA, used full features as the MLP input. There were a total of 59 input features used in method MA. The second method MB used pre-processed data as the MLP input. In method MB, the PCA method was proposed as an input preprocessing algorithm. PCA was employed to reduce the dimensionality of the data by reducing the hue measurements of oil palm FFB. This technique was considered because the extracted data could have correlated components that might affect MLP learning. PCA managed to eliminate those correlated components while keeping as much variation in the information of the input data as possible. Further information about the theory and applications of PCA can be obtained elsewhere [30]. The full-feature data for the training set were reduced to *m* principal components by using the PCA approach. The optimum *m* was determined based on the highest classification accuracy of test data. The processes of both methods are illustrated in Figure 5.

Basically, the procedures of PCA for this work are as follows. First, the hue measurements were normalized so that they have zero mean and unity variance. Then the covariance of each combination of variables was calculated and stored into a covariance matrix, as below,

(9) $$\sum =\left(\begin{array}{cccc} s_{1}^{2} & s_{12} & \cdots & s_{1q}\\ s_{21} & s_{2}^{2} & \cdots & s_{2q}\\ \vdots & \cdots & \ddots & \vdots \\ s_{N1} & s_{N2} & \cdots & s_{q}^{2} \end{array}\right)$$

The eigenvectors **A**=[**a**_1_, **a**_2_, …, **a***_q_*] of Σ were calculated and arranged in ascending order of eigenvalues **λ**=(λ_1_, λ_2_, …, λ*_q_*). Suppose that **x**=(X_1_, X_2_, …, X*_q_*)' denote an observation of the hue values and **y**=(Y_1_, Y_2_, …, Y*_q_*)' is the derived set of the hue values, then

(10) y=A′x

In this work, the first *m* uncorrelated principal components (PCs) were accounted for using [30]

(11) $$\frac{\sum_{i=1}^{m}\lambda_{i}}{\sum_{i=1}^{q}\lambda_{i}}$$

where the total variation was considered as the new features to be fed into an MLP for classification. The total variations with their corresponding number of PCs are shown in Table 5.

## 3. Results and Discussion

Table 6 shows the results of the method MA. The results show that by using different forms of transfer functions in input and output neurons, the performance of each combination was different due to the different form of mapping in the ANN. The CC combination indicated the highest performance of 91.67%. The combination that implemented the logistic sigmoid function for both input and output neurons managed to classify the ripeness of oil palm FFB when all 59 features were used. Thus, this indicated that for a problem of 59 inputs and 4 ripeness categories, 4 output neurons were able to give the highest classification accuracy. This could be due to distinct binary output representation for each ripeness class, making it easier for the MLP to learn and differentiate the mapping, in comparison to 2 output neurons. Also, each of the 4 neurons has to solve for narrower range of output (*i.e.*, between 0 and 1) in comparison to the case of 1 output neuron (*i.e.*, output ranges within 1 and 4). The difference in the performance of ANNs with different numbers of output neurons could be well explained in the different numbers of unknowns (*i.e.*, different numbers of neuronal outputs to be solved for) as well as the different ranges of output value that each neuron can possibly produce. For instance, with 1 output neuron, it is a 59-to-1 problem (*i.e.*, 59 inputs and 1 output), where the output ranges from 1 to 4 (*i.e.*, 4 classes of ripeness). With 4 output neurons for example, it is a 59-to-4 problem where the ANN has to solve for 4 output values, each being between 0 and 1. Somehow, the results have revealed that different numbers of output neurons with different ranges of output values contribute to the mapping complexity of an ANN, and hence affecting its performance.

In method MB, the number of features was reduced from 59 hue measurements to 12 different numbers of PCs. These numbers were determined based on the total variation obtained by the method explained in Section 2.3. The performance of MLP for every combination and every number of PCs are shown in Table 7. The CC combination with 5 PCs yielded the best performance of 91.67% correct classification.

**Figure 6.**
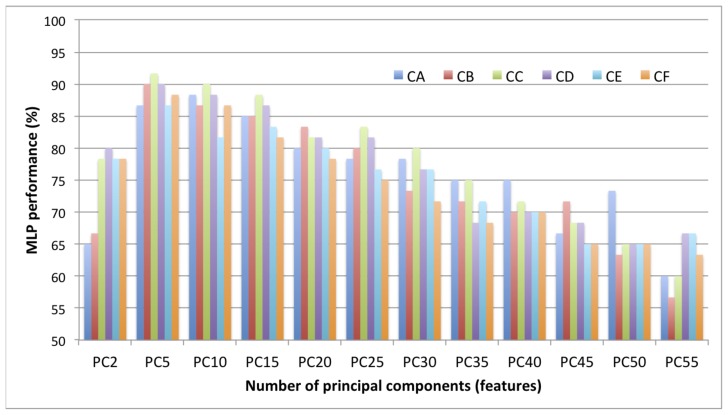
MLP performance based on number of features.

Figure 6 illustrates the overall MLP performance for method MB. When the number of features was reduced from 59 to 55, the MLP's classification accuracy decreased to less than 70%. The accuracy started to increase as the number of PCs was reduced until it reached more than 80% at 5 to 15 PCs. This is due to elimination of correlation in the data, whose existence may confuse the MLP learning process. Yet, MLP was unable to classify the oil palm FFB images correctly when there were only 2 PCs because there might not be enough information for the MLP to learn.

**Figure 7.**
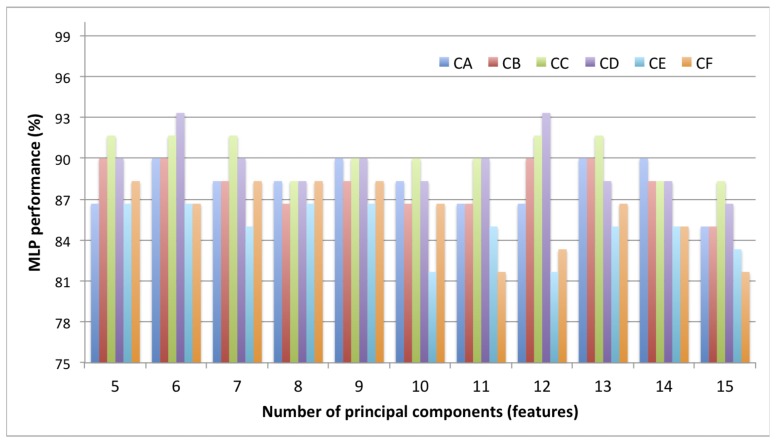
MLP performance based on number of features (5–15 PCs).

To find the optimal number of features, MLP performance was further investigated for 5 PCs to 15 PCs. The results are shown in Table 8 and Figure 7. It was found that at CD combination of 6 and 12 PCs, the MLP performance was 93.33%, which indicated the highest performance.

In comparison to method MA, method MB reduced the number of features by finding the minimum number of features required for an MLP to give the best classification accuracy. It is proven in this work that even though the features were reduced, MLP based on method MB managed to classify oil palm FFB images with higher classification accuracy than method MA. By reducing the number of features, the number of input neurons for MLP is reduced. Thus, the MLP architecture becomes simpler. This could reduce memory requirement for execution of the task and provide faster classification.

As discussed in the Introduction section, conventional oil palm FFB harvesting method involves observing the number of loose fruits and the color of the FFB surface. Such method is subjective and tends to be erroneous. Besides, it takes so much time for a harvester to count loose fruits before deciding whether to cut off an oil palm FFB. By using the proposed classification system that employed MLP and PCA, the subjective and time-consuming judgment of human grading could be solved.

Even though there have been similar image sensor studies to classify the ripeness of oil palm FFB, most of them implemented laboratory setup and used controlled lighting when capturing the FFB images. The classification accuracy of such methods reached up to 100% [18]. Meanwhile, Jamil *et al.* predicted that the classification accuracy would drop when the setup were to be changed to outdoor environment due to variance in illumination. Later, they came up with a neuro-fuzzy technique to grade oil palm FFB in outdoor environment and reported a classification accuracy of only 73.3%. In comparison, for FFB images captured in outdoor environment, this proposed work employing a simple MLP gives a higher ripeness classification accuracy of 93.33% than the neuro-fuzzy approach. Therefore, the proposed approach would be beneficial for automated ripeness decision during harvesting process in determining whether an FFB should be cut off from an oil palm tree.

## 4. Conclusions

The algorithm for the ripeness classification of oil palm FFB has been successfully implemented. The performance of MLP has been investigated for classification purpose by using data with either full features or reduced features. In the first method, all 59 hue measurements from segmented fruit images were used as the features to characterize the oil palm FFB ripeness. MLP managed to classify the ripeness of oil palm FFBs with 91.67% correct classification. In the second method, PCA was used to obtain a number of principal components that represented the new features to be fed into MLP. By using only 6 features, MLP managed to classify the ripeness of oil palm FFB with 93.33% correct classification. Results indicated that even though the features were reduced, the best classification performance improved by 1.66%. This method seems effective in improving MLP performance. Besides, training with reduced features decreases the computational time by reducing the number of MLP inputs. In conclusion, the developed ripeness classifier can serve as a color sensor for automated oil palm FFB ripeness classification, in order to expedite the accurate ripeness grading during a harvesting process.

## Figures and Tables

**Table 1.** Properties of each investigated MLP neural network.

**Combination Label**	**Transfer Function (Hidden Neuron)**	**Transfer Function (Output Neuron)**	**No. of Output Neurons**
CA	logsig	logsig	4
CB	tansig	logsig	4
CC	logsig	logsig	2
CD	tansig	logsig	2
CE	logsig	purelin	1
CF	tansig	purelin	1

**Table 2.** Output coding representations for CA and CB combinations.

**Ripeness Class**	**MLP Output**			
	**1**	**2**	**3**	**4**
Unripe	1	0	0	0
Underripe	0	1	0	0
Ripe	0	0	1	0
Overripe	0	0	0	1

**Table 3.** Output coding representations for CC and CD combinations.

**Ripeness Class**	**MLP Output**		
	**1**	**2**	
Unripe	0	0
Underripe	0	1
Ripe	1	0
Overripe	1	1

**Table 4.** Output coding representations for CE and CF combinations.

**Ripeness Class**	**MLP Output**
Unripe	1
Underripe	2
Ripe	3
Overripe	4

**Table 5.** Total variation with its corresponding number of PCs.

Total variation	0.2	0.0482	0.06	0.0014	0.00031	0.00019	0.000123
Number of PCs	2	5	10	15	20	25	30
Total variation	0.00008	0.000046	0.00003	0.000017	0.000008		
Number of PCs	35	40	45	50	55		

**Table 6.** Oil palm FFB ripeness classification accuracy for method MA.

**Combination Label**	**Classification Accuracy (%)**
CA	88.33
CB	88.33
CC	**9**1.67
CD	90.00
CE	85.00
CF	86.67

**Table 7.** Oil palm FFB ripeness classification accuracy for method MB.

	**No. of PC (Features) and Performance (%)**											
	**2**	**5**	**10**	**15**	**20**	**25**	**30**	**35**	**40**	**45**	**50**	**55**
CA	65.00	86.67	88.33	85.00	80.00	78.33	78.33	75.00	75.00	66.67	73.33	60.00
CB	66.67	90.00	86.67	85.00	83.33	80.00	73.33	71.67	70.00	71.67	63.33	56.67
CC	78.33	**9**1.67	90.00	88.33	81.67	83.33	80.00	75.00	71.67	68.33	65.00	60.00
CD	80.00	90.00	88.33	86.67	81.67	81.67	76.67	68.33	70.00	68.33	65.00	66.67
CE	78.33	86.67	81.67	83.33	80.00	76.67	76.67	71.67	70.00	65.00	65.00	66.67
CF	78.33	88.33	86.67	81.67	78.33	75.00	71.67	68.33	70.00	65.00	65.00	63.33

**Table 8.** Oil palm FFB ripeness classification accuracy for method MB (5–15 PCs).

	**No. of PC (Features) and Performance (%)**										
	**5**	**6**	**7**	**8**	**9**	**10**	**11**	**12**	**13**	**14**	**15**
CA	86.67	90.00	88.33	88.33	90.00	88.33	86.67	86.67	90.00	90.00	85.00
CB	90.00	90.00	88.33	86.67	88.33	86.67	86.67	90.00	90.00	88.33	85.00
CC	91.67	91.67	91.67	88.33	90.00	90.00	90.00	91.67	91.67	88.33	88.33
CD	90.00	**9**3.33	90.00	88.33	90.00	88.33	90.00	**9**3.33	88.33	88.33	86.67
CE	86.67	86.67	85.00	86.67	86.67	81.67	85.00	81.67	85.00	85.00	83.33
CF	88.33	86.67	88.33	88.33	88.33	86.67	81.67	83.33	86.67	85.00	81.67

## References

[1] Brosnan T., Sun D.W. (2004). Improving quality inspection of food products by computer vision—A review. J. Food Eng..

[2] Corley R.H.V., Tinker P.B. (2003). The Oil Palm.

[3] Vaughan J., Nicholson B., Geissler C., Dowle E., Rice E. (2009). The New Oxford Book of Food Plants.

[4] Hitam A.H., Yusof A.M., Basiron Y., Jalani B., Chan K. (2000). Mechanization in Oil Palm Plantations. Advances in Oil Palm Research.

[5] Jalil A. Grading of FFB for Palm Oil Mills in Malaysia.

[6] Malaysian Palm Oil Board (2003). Oil Palm Fruit Grading Manual.

[7] Ismail W.I.W., Bardaie M.Z., Hamid A.M.A. (2000). Optical properties for mechanical harvesting of oil palm FFB. J. Oil Palm Res..

[8] Ghazali K.H., Samad R., Arshad N.W., Karim R.A. Image Processing Analysis of Oil Palm Fruits for Automatic Grading.

[9] Choong T., Abbas S., Shariff A., Halim R., S M.H., Yunus R., Ali S., ahmadun F.R. (2006). Digital image processing of palm oil fruits. Int. J. Food Eng..

[10] Alfatni M.S.M., Shariff A.R.M., Shafri H.Z.M., Saaed O.M.B., Eshanta O.M. (2008). Oil palm fruit bunch grading system using red, green and blue digital number. J. Appl. Sci..

[11] Hudzari R., Ishak W.W., Noorman M. (2010). Parameter acceptance of software development for oil palm fruit maturity prediction. J. Softw. Eng..

[12] Jaffar A., Jaafar R., Jamil N., Low C.Y., Abdullah B. (2009). Photogrammetric grading of oil palm fresh fruit bunches. Int. J. Mech. Mechatron. Eng..

[13] Guan L.C. (2005). Stepwise Discriminant Analysis on Oil Palm Fruit's Hues for Ripeness Grading Using Machine Vision System. M.Sc. Thesis.

[14] Tan Y.A., Low K.W., Lee C.K., Low K.S. (2010). Imaging technique for quantification of oil palm fruit ripeness and oil content. Eur. J. Lipid Sci. Technol..

[15] Ismail W.I.W., Razali M.H. (2010). Hue Optical Properties to Model Oil Palm Fresh Fruit Bunches Maturity Index.

[16] Zakaria A., Shakaff A.Y.M., Masnan M.J., Ahmad M.N., Adom A.H., Jaafar M.N., Ghani S.A., Abdullah A.H., Aziz A.H.A., Kamarudin L.M. (2011). A biomimetic sensor for the classification of honeys of different floral origin and the detection of adulteration. Sensors.

[17] Zhang Y., Wu L. (2011). Crop classification by forward neural network with adaptive chaotic particle swarm optimization. Sensors.

[18] Junkwon P., Takigawa T., Okamoto H., Hasegawa H., Koike M., Sakai K., Siruntawineti J., Chaeychomsri W., Sanevas N., Tittinuchanon P. (2009). Potential application of color and hyperspectral images for estimation of weight and ripeness of oil palm (Elaeis guineensis Jacq. var. tenera). Agric. Inf. Res..

[19] Jamil N., Mohamed A., Abdullah S. Automated grading of palm oil Fresh Fruit Bunches (FFB) using neuro-fuzzy technique.

[20] May Z., Amaran M.H. (2011). Automated oil palm fruit grading system using artificial intelligence. Int. J. Eng. Sci..

[21] Chinchuluun R., Lee W.S., Bhorania J., Pardalos P.M. (2009). Clustering and classification algorithms in food and agricultural applications: A survey. Advances in Modeling Agricultural Systems.

[22] Gonzalez R., Woods R. (2010). Digital Image Processing.

[23] Abdullah M., Guan L., Mohamed A., Noor M. (2002). Color vision system for ripeness inspection of oil palm elaeis guineensis. J. Food Process. Pres..

[24] Ayrulu-Erdem B., Barshan B. (2011). Leg motion classification with artificial neural networks using wavelet-based features of gyroscope signals. Sensors.

[25] Baladrón C., Aguiar J.M., Calavia L., Carro B., Sánchez-Esguevillas A., Hernández L. (2012). Performance study of the application of artificial neural networks to the completion and prediction of data retrieved by underwater sensors. Sensors.

[26] Jeon H.Y., Tian L.F., Zhu H. (2011). Robust crop and weed segmentation under uncontrolled outdoor illumination. Sensors.

[27] Rafiq M., Bugmann G. (2001). Neural network design for engineering applications. Comput. Struct..

[28] Abdullah M., Mohamad-Saleh J., Fathinul-Syahir A., Mohd-Azemi B. (2006). Discrimination and classification of fresh-cut starfruits (Averrhoa carambola L.) using automated machine vision system. J. Food Eng..

[29] Priddy K.L., Keller P.E. (2005). Artificial Neural Networks: An Introduction.

[30] Jollife I. (2002). Principal Component Analysis.

